# Ticking time bomb: implications of the COVID-19 lockdown on e-waste management in developing countries

**DOI:** 10.14324/111.444/ucloe.000023

**Published:** 2021-08-04

**Authors:** Oluwadamilola A. Adejumo, Olubisi F. Oluduro

**Affiliations:** 1 Lecturer, Department of Business Law, Faculty of Law, Obafemi Awolowo University, Ile-Ife, Nigeria; 2 Senior Lecturer, Department of Public Law, Faculty of Law, Obafemi Awolowo University, Ile-Ife, Nigeria

**Keywords:** coronavirus, COVID-19 lockdown, developing countries, e-waste, e-waste management, non-physical interactions

## Abstract

The COVID-19 pandemic has altered the course of events globally since the outbreak of coronavirus disease (COVID-19) in late 2019 giving further credence to the long-standing belief that the world is indeed a global village. There have been different responses by countries to the raging pandemic including the imposition of lockdowns, quarantine and isolation. The imposition of lockdowns, whether full or partial, has not been without major consequences, which has led to information, communication and technological (ICT)-based measures to minimise the effect of the lockdown and as an alternative to physical interactions. The use of ICT devices to bridge the gaps created by the lockdown on schools, businesses and other sectors has led to the increased use of electronic devices. The challenge of electronic waste (e-waste) management in developing countries has been around for a while and the increased use of electronic devices is likely to compound the challenge during and post COVID-19. Whilst the development of ICT-based options as viable alternatives to face-to-face interactions may not be a negative development, this article argues that the existing frameworks are inadequate to manage the resultant increase in e-waste in most developing countries and that there is need now more than ever before for developing countries to exercise caution in embracing these ICT-based options without putting in place measures to ensure that there is increased capacity to manage and dispose of the e-waste created.

## Introduction

The COVID-19 pandemic has altered the course of events globally since the outbreak of coronavirus disease (COVID-19) in late 2019 in Wuhan, China. This event has further given credence to the long-standing notion that the world is indeed a global village. The virus has spread across the world, leading to COVID-19 being declared a global pandemic by the World Health Organization (WHO) in March 2020 [1]. As of 20 January 2021, a total of 94,963,847 cases has been recorded with approximately 2,050,857 deaths recorded globally [2]. Different countries have had different responses to the raging pandemic including the imposition of lockdowns, quarantine and isolation. Rwanda was the first African country to impose a national lockdown followed by other African countries imposing either total or partial lockdowns [3]. The imposition of the lockdown, whether full or partial, has not been without major consequences impacting on almost every sector of the economy including the educational, aviation, health and environmental sectors. The use of information, communication and technological (ICT) measures to minimise the effect of the lockdown and as an alternative to face–face activities has increased significantly. The use of ICT devices to bridge the gaps created by the lockdown on schools, businesses and other crucial sectors has led to the increased use of electronic devices that were initially considered optional or a luxury. The increased use of these devices is not without consequences on the environment. Some of the likely challenges are the increased importation of electronic waste (e-waste) in the form of used electronic devices and the challenge of disposal and management of these devices once they reach their end-of-life stage and become waste. The challenge of e-waste management in developing countries has been ongoing for a while and has yet to reach a decisive stage in terms of the resolution of the challenge. Thus, the increased use of devices is likely to compound the challenge.

The aim of this article, therefore, is to discuss the environmental impact of the COVID-19 pandemic on developing countries, highlighting the challenges inherent in the likely increase in the use of electronic and ICT options during the pandemic.

The first part of the article provides some background information on COVID-19. The second part discusses the implication of the pandemic and the impact of the lockdown on usual activities whilst emphasising the increased reliance on ICT devices replacing usual physical interactions. The article argues that the lockdown has both positive and negative options, which, if not adequately controlled and planned for, will create increased e-waste that most developing countries in Africa lack the capacity to manage effectively. The third part provides an overview of e-waste and discusses e-waste as a challenge most developing countries are faced with. The fourth part of the paper highlights existing legal and institutional frameworks to manage e-waste, highlighting the challenges inherent in these frameworks. The fifth part discusses the way forward for developing countries and the final part concludes that the development of ICT options, as viable alternatives to face-to-face interactions, may not be a negative development. However, the existing frameworks are inadequate to manage the resultant increase in e-waste and there is a need for measures to minimise the generation of such waste and its proper disposal during and post-COVID-19 lockdown.

## The COVID-19 pandemic

COVID-19 is a novel global pandemic caused by severe acute respiratory syndrome coronavirus (SARS-CoV-2), one of the large family of viruses causing severe diseases, such as the Middle East respiratory syndrome (MERS), and severe acute respiratory syndrome (SARS) [4]. It originated in Wuhan, China around November 2019, spreading quickly across other parts of the globe in less than six months [5]. On 30 January 2020, the WHO declared that the outbreak constituted a Public Health Emergency of International Concern (PHEIC) [6]. It has now affected about 16 million people, with more than half a million fatalities globally. It is transmitted primarily from person to person, through droplets from coughing or sneezing or by an infected person speaking to others around him who inhale these droplets – community transmission. It is also airborne and can be contracted through touching contaminated surfaces such as doorknobs and money and people touching their faces [4]. Its incubation period is between 2 and 14 days, with symptoms including dry cough, fever, fatigue, loss of smell and taste, as well as shortness of breath, sore throat, skin rashes or discolouration of fingers or toes, and nasal congestion, leading to difficulty in breathing. It may, however, be spread before or without manifestation of any symptoms by asymptomatic carriers. It may start off as a mild-to-moderate illness but can progress to serious cases including blood clots and multi-organ failure. Some may not require hospitalisation, but people with underlying medical conditions such as diabetes, high blood pressure, cancer, heart and lung problems, etc., particularly adults in the upper age bracket, 50 years and above, may be at higher risk and the illness may result in death or serious health implications after treatment [4].

Research is ongoing on the exact cause of the virus, but it is believed that bats are the mutation agents for the virus [7], which undergoes certain mutations that can then be transmitted to humans. Infection figures vary from country to country around the world, with the United States (US) leading the fray; daily infection figures are still on the rise. The US alone accounts for almost a quarter of the global figures [8], while on the African continent, which has the lowest infection rates among other regions of the world, South Africa has the highest infection rates [8]. Africa’s low infection rates remain baffling to the rest of the world. Preventive measures such as social distancing, frequent hand washing with water and soap, or rubbing hands with alcohol-based hand sanitisers, and use of face masks in public settings are some of the measures to curtail the spread of the virus.

Although the impact of the outbreak of the virus has been largely negative, particularly in the context of public health and safety, some positive implications of the outbreak on the environment have been recorded. The reduction in industrial activity, tourism, travel and flights across the globe has actually had a positive impact on the environment resulting in the reduction of the release of pollutants. Indeed, some countries reported a drop in carbon emissions [9]. These developments may, however, be considered temporary as there is a likelihood that these temporary gains may be lost once the tide of the raging pandemic abates and most of these activities return to the status quo. Despite these temporary gains, there is a need to focus on likely dangers that the pandemic portends to the environment now and in the near future.

## Implications of COVID-19 lockdowns and increased reliance on ICT alternatives to physical interactions

As part of measures to ensure social distancing and reduce the spread of COVID-19, lockdowns were imposed in countries across the world. Mitra et al. in their study reported that transmission of COVID-19 is via airborne droplets and that, to effectively control its spread, there is a need to reduce the concentration of people in public meeting places [10]. Indeed, this appears to be the rationale for the imposition of either total or partial lockdowns by most countries in response to the outbreak of COVID-19.

The lockdown led to closure of schools, hospitals, offices and businesses and reduced local business travel. Only a few essential services stayed operating. The closure of schools, for instance, meant that pupils stayed at home and measures were put in place to ensure they were engaged while at home. Toquero reported that higher educational institutions were closed in about 188 countries across the world as of 6 April 2020 owing to the vulnerability of school settings to the spread of the COVID-19 [11]. The closure led to the introduction of online systems of learning that most schools were not prepared for although some countries such as Hong Kong and China had adopted an online mode of teaching when schools had to be closed during the outbreak of SARS in 2003. Mitra et al. also corroborated the fact that teaching and assessment of students moved online to an untested and unprecedented scale [10]. This pattern is likely to continue even after the pandemic as there is a strong likelihood that e-learning will continue to complement traditional learning patterns.

Apart from the closure of schools, the imposition of local or national lockdowns also resulted in the closure of some health services that were deemed non-essential [12]. The increasing number of COVID-19 cases has caused a huge strain on the health care systems in most countries with facilities and personnel required for the management and treatment of patients becoming stretched. Also, the fact that health care workers (HCWs) are exposed to a high risk of infection by COVID-19 patients in the course of treatment of these patients [13] made hospital visits to seek care for other ailments undesirable.

The rate of infection of HCWs across the globe is becoming increasingly worrisome. In Africa alone, in May 2020 the WHO reported the infection of 945 health workers in 28 countries, with South Africa having the highest number of infected health workers [14]. An updated report released on 26 May 2020 revealed that Nigeria recorded the highest number in the region with 606 health workers having been infected with the coronavirus [15]. This has made resorting to non-physical visits to hospital for consultation and treatment a preferred option via telemedicine channels. Vidal-Alaball et al. [16] posit that telemedicine is capable of providing support to the healthcare systems in the midst of this pandemic whilst advocating for its continued use even after the pandemic ends. They reported that countries such as China, South Korea, Spain, the US, Japan and many European countries are at different stages of experimenting with and implementing telemedicine. O’Leary also reported the development of various applications (apps) to slow the spread of COVID-19. The apps are used to track the coronavirus or to check symptoms. All these apps require a smart phone to use them as the app uses information from the user’s phone to determine if they have been in contact with any person with the virus [17].

In the same vein, religious activities have also largely moved online with services and prayer meetings being conducted on different online platforms globally. Places of worship recognised the spiritual needs of the faithful and created access to their programmes through social media platforms holding livestream worship on Facebook, Twitter, YouTube, websites and other platforms [18]. E-commerce, contact-free buying and selling and business meetings have also thrived in a bid to ensure social distancing. The 2020 Annual General Meeting (AGM) of the Nigerian Bar Association, for instance, was a strictly online event and was the first ever virtual AGM to be held by the Association [19]. All these virtual events and activities will require the use of one form of electronic device or the other.

Goldschmidt reported a growing reliance on the use of technology to learn, live and stay connected. The study placed emphasis on the use of technology to leverage and maintain social, physical, emotional and even spiritual wellbeing for children as the closure of parks, playgrounds and other social services centres were not uncommon during the pandemic [18], with children having to rely largely on the use of devices for play and entertainment.

Jiang et al. reported on the extra energy demands in their study arising from energy consumed by confinement measures including working from home and telemedicine during the pandemic [20]. They specifically attributed the increased energy demand to increased use of ICT devices to support digitalisation and teleworking during the lockdown in both developed and developing countries.

Although the lockdown has come with some gains for the environment with reports of significant reduction in emissions and pollution [10,21], the lockdown also comes with looming challenges for the environment if adequate plans are not put in place to address them. O’Leary particularly notes that there is a strong likelihood that business, organisations, culture and society will be forever changed by all the adaptations to the pandemic as solutions generated by the information systems and technology communities will be used even in future settings and post-COVID-19 pandemic [17].

## The e-waste challenge in developing countries

E-waste consists of damaged or outdated electronic devices, products, materials and spare parts. They are electronic devices that are discarded or dumped by the owners or buyers after the end of their useful life, or because of the rapid expansion of technology, or consumption change in society. These may not necessarily be outrightly damaged, but may be refurbished, reused (working and repairable electronics), resold, refined or salvaged through recycling.

The world is currently generating e-waste faster than it can be recycled or repurposed. Electronic waste will increase to 52 million metric tonnes by 2021, and 120 million tonnes yearly by 2050, of which only 20% would be recycled [22,23]. As a result of this, e-waste is generally imported, mostly illegally to the developing countries of Africa from the developed countries of Europe, America and Asia, notwithstanding the ban on the same through various international conventions [24–28]. E-waste is peculiar in that it is known to contain hazardous substances such as toxic metals and organic chemicals, which require due care in handling, failing which they can cause an array of diseases in humans who handle them inappropriately. Such diseases include genomic, respiratory, neurodevelopment, reproductive and hormonal systems [29]. Waste management has been the bane of many countries, specifically electronic waste otherwise called end-of-life-electronics equipment (EEE).

Throughout much of human history, waste has always had an inherent value [30], yet waste management is one of the greatest environmental challenges, as enormous amounts of waste are generated daily resulting in gargantuan annual volumes globally, running to more than 50 million metric tonnes of defunct electronic products. The higher percentage of these are classified as hazardous because they contain toxic chemicals.

Used electronic equipment is dumped on the developing countries daily by the developed countries in large amounts [31]. An unfortunate aspect of this is that the former lack the requisite technologies and techniques for processing e-waste. Consequently, primitive recycling technologies are employed by these countries, which expose people to significant health risks through contact with a range of hazardous substances contained within such as lead, brominated flame retardants, chromium, lithium, mercury, cadmium and polychlorinated biphenyls (PCBs).

Nigeria’s fast-growing second-hand computer industry contributes to large-scale computer waste importation, with the nation’s ports handling an estimated 500 container loads per month, with each container carrying about 500,000 used computers and other electronic equipment from the USA, Europe and Asia. Lagos, the commercial capital, harbours two large second-hand electronics markets – Alaba International Market and the Computer Village – with up to 500 tonnes coming in daily, mostly shipped as used electronics [32]. It is not uncommon in Nigerian markets to find people marketing used products from the UK as a preferred option to brand-new electronic products that are believed to be of less durable quality. Colloquial terms such as ‘UK used’ are commonly used in marketing second-hand mobile phones and laptops that have not had much use.

The cost of recycling e-waste in developed countries also encourages the shipment of used electronic equipment to developing countries that have a thriving market for this equipment and which have to deal with the responsibility of disposing of them once they eventually stop working. Most of these devices end up being disposed of improperly by people resorting to primitive recycling methods. Fehm notes that e-waste recycling is not only very expensive but it is also labour intensive as the parts are ordinarily not intended to be taken apart [33]. The process of recycling e-waste requires expensive machinery and sophisticated procedures which most developing countries do not have. The resort to primitive recycling methods by developing countries may cause serious human health hazards, particularly in children who are more vulnerable. For example, their functional systems such as the central nervous, immune, reproductive and digestive systems, which are still developing, may be hampered by exposure to toxic substances. This damage might be irreversible.

Recycling is a form of disposal of e-waste in Nigeria that has sprung up in Lagos and a couple of other cities. Although largely done informally, it is focused on selected valuable materials. Participants are either itinerant waste buyers or scavengers who target valuable materials such as plastics, central processing units (CPUs), batteries, screens, glass and metals. Their activities can have an impact on the reduction of the net volume of disposed e-waste. Waste burning is also common in Nigeria. Burning of waste has a well-documented association with the incidence of respiratory health symptoms among adults and children. Burning of toxic waste produces chemicals such as dioxins, furans and dioxin-like polychlorinated biphenyls, which are suspected carcinogens that damage the nervous and immune systems and are very harmful even in miniscule quantities [34,35].

Landfills are a standard method for disposing of waste in the developed and developing countries. However, improperly built landfills can cause environmental and human health problems. Just as densely packed organic matter produces methane as it rots, toxic waste can cause even more devastation as it can cause explosions. Bacteria working on this waste can break down waste acids and particulates which can be concentrated enough to dissolve poisonous heavy metals such as lead and cadmium among others. Water leaching through the landfill can carry such toxins into the groundwater or nearby bodies of water and from there into drinking water and thus into the food chain [36]. The Olusosun landfill in Lagos is Africa’s largest and the world’s fourth largest landfill site, covering an estimated 43 hectares, and is 18 m deep. It has been in existence for several decades with no plan to transform or close it. Aside from being a beehive of activities to hordes of daily scavengers looking for disposed waste items, it is also a place of accommodation to many (scavengers), who work at the site and live in a tented village on top of the trash. Besides, it is hedged in between the residential part of the bustling city, where about 5 million people live in a 10 km radius surrounding the site [32,37]. The site is ever-burning, releasing toxic fumes into the air with a blanket of soot over the area, affecting both the scavengers and residents. Diseases such as skin irritation, dysentery, water-related diseases, paralysis and nausea are reported by residents living within a 3 km radius of the site [38].

A large percentage of African countries are poor with the greatest proportion of the people living off the environment, directly or otherwise attached to the land and the resources from it [39]. Coupled with this is the state of awareness of the population, as thousands of the scavengers are basically unaware of how dangerous their work is. This accounts for why they dismantle items that contain mercury, lead, beryllium and lithium, among others, with their bare hands, which constitutes a great risk to their health. These could lead to adverse health conditions such as inflammation and oxidative stress that could aggravate to cardiovascular diseases, chronic kidney diseases, cancer and DNA damage [40].

Arising from the pandemic, there have been reports of the challenges of waste management, particularly the management of bio-medical wastes in developing countries [41]. Changes in waste disposal patterns have also posed a huge challenge to waste management during the pandemic, especially in developing countries. Oyedotun et al. specifically highlighted the possibility of cross-contamination between residents and landfill scavengers who are dismantling waste during the pandemic [42].

## Legal and institutional framework for the management of e-waste and their challenges

### International and regional frameworks

The first international attempt to address the exportation of e-waste from developed to developing countries was made under the Basel Convention on the Control of Transboundary Movements of Hazardous Wastes and their Disposal that was adopted in 1989 [24]. The Convention specifically addresses the vulnerability of the least developed countries (LDCs) due to the increasing likelihood of businesses from industrialised states disposing of their hazardous waste in cheaper as well as less environmentally regulated countries and promotes observance of environmentally sound waste management principles [24]. The USA, which is one of the largest exporters of hazardous waste, has only signed but has not ratified the Basel Convention and continues to export hazardous waste, and in particular e-waste, to the LDCs. McAllister et al. notes in this context that the people most severely affected by e-waste are effectively unrecognised by those who are most responsible for generating the waste [43].

The Bamako Convention came as a follow-up to the Basel Convention in 1991 [25]. The Convention is a regional response by African countries to address the issue of the importation of hazardous waste to African countries from developed countries [44]. Initially negotiated by 12 countries, the Bamako Convention currently has been ratified by 25 countries and the Convention is considered to be stronger than the Basel Convention in its prohibition of the importation of hazardous waste as it allows no exceptions [25,44]. Obligations under the Bamako Convention include ensuring a minimum reduction in the generation of hazardous waste as well as the prevention and minimisation of the effect of pollution in the handling of hazardous wastes [25].

Illegal dumping of e-waste by whatever means on the developing nations that have not the requisite technology to recycle the same also constitutes a negation of Article 191 of the Treaty on the Functioning of the European Union (TFEU), which provides for the protection of human health, one of the objectives of the Community’s policy on the environment. The policy, which aims at a high level of protection, is based on a precautionary principle, the requirements of which must be integrated into the definition and implementation of other Community policies. It further follows from the case law of the European Court that the precautionary principle may also apply in policy on the protection of human health, which, according to Article 168 TFEU, likewise aims at a high level of protection [45]. In the Wallonian case [46], the Court considered a Wallonian ban on the import of waste from anywhere outside the region of Belgium on the basis of the proximity principle, namely that waste should be disposed of as close to the place of production as possible. This is to avoid environmental costs and risks of transporting the waste, and it establishes a principle of environmental equity. Clean places are not to bear the environmental costs generated by dirty places. Contrastingly, the court in the Dusseldorp case [47], an application to export two loads of oil filters for processing, was refused by the Dutch authorities on the grounds that, under Dutch law, export of waste for recovery was only permitted if there were superior processing techniques abroad, or there was insufficient capacity in the Netherlands. The Court of Justice noted that such an export restriction provided an advantage for national facilities. The whole idea behind dumping e-waste on developing countries where these goods may be subsequently processed in dangerous and inefficient conditions, as obtained more particularly in the West African sub-region, harming the health of local people and damaging the environment, negates substantially the provisions of the Basel Convention and the Organisation for Economic Co-operation and Development (OECD) rules on hazardous and toxic wastes, to which some of these developed countries are party [31].

Various international calls for action in recent times include the Libreville Declaration, being the first Inter-Ministerial Conference on Health and the Environment in Africa in 2008, the Busan Pledge for Action on Children’s Environmental Health 2009 and the Strategic Approach to Integrated Chemical Management’s expanded Global Plan of Action ICCMM3 2012 [26]. The world’s first forum on e-waste was convened in 2006 at the 8^th^ meeting of the Conference of the Parties (CoP) to the Basel Convention, leading to the Nairobi Declaration on creating innovative solutions for the environmentally sound management of electronic waste. However, this does not seem to have any form of bearing or influence on many of the developing countries’ take on the subject matter as illegal trading in it grows unabated, to the detriment of their environment and the people’s health.

### National frameworks and challenges of e-waste management in some developing countries

Apart from the efforts noted above at international and regional levels, countries have also made efforts to combat the e-waste challenge. In developed countries, there are advanced approaches to e-waste management that help to regulate, to a large extent, e-waste management. Approaches such as the European Union Extended Producer Responsibility (EPR), the Consumer Pays Model and charging of Advanced Recycling Fees at point of purchase have been somewhat effective. Some developed countries have also made significant efforts to adopt upstream recycling methods and have the infrastructure and expertise for upstream recycling of e-waste. However, this is not the case in developing countries. Gamaralalage and Premakumara note that the progress in the practical implementation of national regulations on e-waste at the local level is limited and challenging [48]. Existing frameworks in some developing countries are discussed below. (See Table 1 for summary of existing frameworks and challenges identified in some developing countries.)

**Table 1.** Summary of e-waste management framework in some developing countries and identified challenges

Country	Framework for e-waste Management	Challenges
Nigeria	Harmful Toxic Waste (Special Criminal Provision) Act 1988 National Environmental (Sanitation and Wastes Control) Regulations 2009 National Environmental (Electrical/Electronic Sector) Regulations 2011 Guide for Importers of Used Electrical and Electronic Equipment into Nigeria Nigerian Communications Commission (NCC) E-waste Regulations for players in the Communications Industry 2019	Weak enforcement mechanisms Poor implementation of the extended producer responsibility programme Poor public awareness and sensitisation Porous borders allowing for smuggling of e-waste Thriving informal recycling sector owing to poverty and poor economic conditions Unattractive environment for investment in formal recycling projects
Ghana	Prohibition on Manufacture, Sale and Importation of Incandescent Filament Lamps, Used Refrigerators, Used Refrigerators-Freezer, Used Freezers and Used Air Conditioners Regulations, LI 1932 (2008) Hazardous and Electronic Waste Control Management Act 917 of 2016 Hazardous, Electronic and Waste Control and Management Regulations (2016)	Thriving informal recycling sector owing to poverty and poor economic conditions Unregulated informal sector Poor incentives for compliance with EPR programme
Kenya	National Environmental Management Authority (NEMA) Guidelines for e-waste management, 2010 Information Technology (ICT) Policy by the Ministry of ICT	Poor public awareness of negative impact of e-waste Absence of needed capacity to implement and enforce policies Poor waste collection practices that do not allow for segregation of e-waste and thriving informal e-waste handling owing to the prevalence of poverty
India	E-Waste Management and Handling Policy 2011 E- Waste (Management) Amendment Rules (2018)	Illegal importation Crude processing and recycling arising from a lack of finance and infrastructure for formal recycling Defective legislations with no penalty for non-compliance to have deterrent effect Poor public awareness on proper handling and disposal of e-waste
China	Circular Economy Promotion Law Administrative Rules on Prevention of Pollution by WEEE 2008 Collection and Treatment Decree on Waste Electrical and Electronic Equipment 2011 EPR Policy 2012	Lack of regulation for the informal sector Lack of infrastructure for formal recycling Fragmentation of existing laws Weak enforcement efforts

The state of Nigeria’s legal and policy disposition to hazardous waste is parlous and unexpected to say the least, considering that the nation was jolted into consciousness of the environment by the dumping of hazardous waste in Koko in 1988 by Gianfranco Raffaeli, an Italian businessman [49]. This incident brought about Nigeria taking major steps at fixing its environmental issues with the promulgation of the Harmful Toxic Waste (Special Criminal Provision) Act [50], establishment of the defunct Federal Environmental Protection Agency (FEPA) and a National Policy on the Environment to bring the nation up to date on current environmental trends. Much as that was hoped for, the situation is almost the same today for many obvious reasons, primarily among them being poverty [27]. Thus, for instance, the country cannot be said to be fully ready to effectively handle an increase in the generation of e-waste that is likely to arise during and after the COVID-19 pandemic. The need to bridge the ‘digital divide’ to meet technological needs and the demands of technological advancements will continue to encourage illegal importation of waste electrical/electronic equipment (WEEE) in the name of ‘used’ electrical products.

The Harmful Toxic Waste Act promulgated after the Koko incident was specifically directed at addressing the importation of toxic waste. The Act prohibits the importation, carrying, deposition and dumping of harmful waste on any land, territorial waters and matters relating thereto. Although the penalty for commission of the above offence under the Act is life imprisonment, there has been little success so far. The very porous borders of the country have made the effect of the provision largely redundant as WEEE is still finding its way into the country. In the same vein, the Act also requires persons who generate hazardous waste to cause such waste to be treated using acceptable methods. This provision has also remained largely unenforced as most users of electronic equipment at end-of-life stage dispose of the equipment in the same way other wastes are disposed of. Burning of e-waste without regard to the danger to the environment in a bid to extract copper and gold is also not unusual.

The establishment of the National Environmental Standards Regulation and Enforcement Agency (NESREA) in 2007 as a replacement for FEPA brought about some progress in the framework for addressing the e-waste management challenges in Nigeria. The Agency has made a total of 33 regulations for the protection of the environment since its inception. However, only two of these regulations relate to e-waste: the National Environmental (Sanitation and Wastes Control) Regulations 2009 [51] and the National Environmental (Electrical/Electronic Sector) Regulations 2011 [52]. In addition, the Agency has also released a Guide for Importers of Used Electrical and Electronic Equipment into Nigeria. The Guide reiterates the ban on the importation of WEEE and requires all importers to register with the Agency.

The National Environmental (Sanitation and Wastes Control) Regulations 2009 prohibits littering with waste and encourages the segregation of waste and disposal in designated areas. Although the Regulation recognises different categories of waste including health care waste, solid waste and effluent discharge, it fails to expressly recognise e-waste as a distinct category worthy of specific provisions. This was, however, subsequently covered under the National Environmental (Electrical/Electronic Sector) Regulations 2011 discussed below. Nevertheless, provisions relating to toxic and hazardous waste under the Regulations will apply to e-waste. There is an obligation to ensure that every container or package for storing hazardous waste is secured, marked and labelled. Despite the provision of the Regulations, waste segregation and labelling is still not commonly practised in the country.

The National Environmental (Electrical/Electronic Sector) Regulations 2011 make a more detailed attempt to regulate e-waste importation, disposal and management. The Regulations are anchored on the 5Rs of reuse, reduce, recycle, repair and recover and seek to minimise pollution from electronics and electrical equipment from ‘cradle to grave’. The Regulations relate to both new and used electrical and electronic equipment and advocate the principle of extended producer responsibility including a buy-back plan for management and disposal of e-waste. The Regulations impose responsibility not only on product manufacturers but on distributors, retailers and importers to take back end-of-life products from consumers at designated collection centres where manufacturers and producers are to ensure disposal in an environmentally sound manner.

The Regulations prohibit the importation of end-of-life, unusable, unserviceable electronics and cathode ray tubes. They prohibit the burning of e-waste; disposal in landfills and dump sites; and storage on-site for more than one year and impose penalties for any breaches. They require recycling of e-waste at designated recycling centres. In addition, the Nigerian Communications Commission (NCC) in 2019 adopted the E-waste Regulations for players in the communications industry. This is intended to complement NESREA’s Regulations and regulate the life cycle of electronic and electrical equipment and both agencies are expected to work together towards building an effective partnership for the enforcement of environmental laws and regulations [53].

Although the National Environmental (Electrical/Electronic Sector) Regulations are very detailed in their provisions relating to e-waste importation, management and disposal, not much progress has been achieved in terms of effective implementation and enforcement. A recent report revealed shipment of more than 60,000 tonnes of used electrical and electronics equipment into Nigeria annually via Lagos Port alone. This is in addition to shipment from land borders and other ports. Of this number, more than 25% of the shipped equipment can be categorised as being ‘dead on arrival’ and of no use [54].

Concerns were still raised as recently as January 2020 over the dangers of e-waste management challenges and crude recycling practices with the government being urged to create an enabling environment for recycling plants to spring up and function effectively [55]. Other challenges identified by the NESREA in combating the e-waste challenge include poor implementation of the extended producer responsibility programme and poor public awareness on the dangers of e-waste [56].

In Ghana, there was no detailed legislation on management of e-waste until after 2014 [57]. There was, however, the Energy Efficiency (Prohibition on Manufacture, Sale and Importation of Incandescent Filament Lamps, Used Refrigerators, Used Refrigerator-Freezers, Used Freezers and Used Air Conditioners) Regulations, LI 1932 (2008). Although Ghana is also a Party to the Basel Convention, it is yet to be domesticated under its national laws [58]. The enactment of the Hazardous and Electronic Waste Control Management Act 917 of 2016 in Ghana was the beginning of a specific legal regime for the management of e-waste in Ghana. The Act requires manufacturers and importers of electronic equipment to register with the Environmental Protection Agency (EPA) and to pay an e-waste levy expected to cover the cost of effective management of e-waste [59]. There are also the Hazardous and Electronic Waste Control and Management Regulations, LI 2250 (2016).

Nevertheless, the laws appear more focused on the formal sector and neglect the informal sector. The informal sector is thriving as young men without jobs resort to house-to-house buying of e-waste and delivery to dump sites for a fee. The informality of e-waste recycling, which is common in most developing countries, is closely linked to poverty and the poor economic status of the citizens [58]. These informal practices are largely unregulated and difficult to regulate particularly as most of the stakeholders find it very financially rewarding.

Oteng-Ababio and Amankwaa [57] report that attempts to introduce EPR by two companies in Ghana failed woefully as the responses to calls for the return of obsolete products by consumers yielded a very low response rate. This is perhaps because the incentives were not compelling enough. Thus, about 95% of e-waste recycling is being done informally in an unregulated manner and with reports of extortion of the key stakeholders by the authorities who perceive them as persons involved in some form of illegal activities.

In Kenya, the National Environmental Management Authority (NEMA) formulated guidelines for e-waste management in 2010 as part of efforts to manage e-waste effectively by all stakeholders. Similar to the efforts of the NCC in Nigeria to complement the Environmental Agency’s efforts, an ICT policy was also formulated by the Ministry of ICT to ensure that players in the ICT sector have plans to reduce the impact of their ICT infrastructure on the environment before renewal of their licenses by the Ministry [59]. Otieno and Omwenga identified challenges of e-waste management in Kenya to include poor public awareness of the negative impact of e-waste, the absence of needed capacity to implement and enforce policies on the part of regulatory agencies, poor waste collection practices that do not allow for segregation of e-waste and thriving informal e-waste handling owing to the prevalence of poverty [59].

Similar to what happens in developing African countries, China and India did not have any specific legislation to regulate the importation of e-waste until 2000. The E-Waste Management and Handling Policy was not introduced in India until 2011 and the EPR Policy in China was introduced in 2012 [60]. Prior to the 2012 Policy, however, China had introduced the Circular Economy Promotion Law Administrative Rules on Prevention of Pollution by WEEE and Collection and Treatment Decree on Waste Electrical and Electronic Equipment in 2008 and 2011, respectively, to prevent e-waste-related pollution and regulate recycling activities [60]. In 2018, India also introduced the E-Waste (Management) Amendment Rules (2018) [61].

Patil and Ramakrishna recognised the challenges in India to include illegal importation, crude processing arising from a lack of finance and infrastructure for formal recycling, defective legislations with no penalty for non-compliance to have a deterrent effect and poor public awareness on proper handling and disposal of e-waste [60]. Challenges in China similar to Ghana include the lack of regulation for the informal sector, which carries out most of the recycling activities in both countries. A lack of infrastructure for formal recycling, fragmentation of existing laws and weak enforcement efforts were also identified as challenges of e-waste management in China [60].

The situation in Nigeria is not very different from what happens in other developing countries. The fact that most developing countries that have passed laws and made e-waste regulations only have such laws and regulations in principle without clearly spelt out outlines on the practicalities and financial mechanisms for implementing the laws was emphasised by Supian et al. [62]. They identified poor public awareness of the proper handling of e-waste, overall management issues including policy making and implementation, and poor financing as e-waste management challenges faced by most developing countries.

Some developing countries that have succeeded in establishing formal recycling, including Japan and South Korea, also have some critical challenges. Chung and Murakami-Suzuki reported increased illegal export of e-waste to neighbouring countries to circumvent financial liabilities associated with recycling obligations [63].

## The way forward

It appears clear from the above that having a legal framework without clear implementation strategies will not effectively help in combating e-waste management challenges. Imposing restrictions on the import of used electronics may not be feasible owing to porous borders and poor economies where it is difficult to afford brand-new products. The high level of poverty will also continue to foster activities of informal recyclers and e-waste managers. The practice of copying the framework and strategies of developed countries without the financial ability to implement them and without clearly spelt out guidelines for implementation that takes into cognisance practicality and economic realities are bound to fail.

An ideal e-waste legislation should thus be one that includes a complete ban on e-waste import/export; prohibits crude processing and strengthens and equips informal sectors; enforces EPR principles; and encourages formal recycling industry and rewards consumers for their responsible actions [64]. Nnorom and Osibanjo [65] call for adequate and necessary amendment of existing legislations and implementation processes for achieving a higher recycling rate. In carrying out the amendment process, the views of stakeholders, especially the key players in the informal sectors, should be accommodated as much as possible in the planning, implementation and monitoring processes. Thus, Patil and Ramakrishna [60] recommend a legislation that defines the responsibility of all stakeholders including government agencies, enforcement officers, producers, consumers and the informal recyclers.

Apart from legislation, Liu et al. [66] recommends the building of environmentally protected industrial parks where the recycling of e-waste should be performed to alleviate the negative impact of e-waste on the environment. This can be done via public–private partnership [59].

Developing countries should also begin to do research on phytoremediation and bioremediation to transform the environment contaminated by e-waste. This is even more necessary now that there is increased use of electronic equipment and the resultant waste that will be generated over time [67].

There is also an urgent need to promote public awareness of the risks involved in improper handling of e-waste.

Technology development and information exchange on best management practices should be encouraged among developing and developed countries. Stronger commitment should be made by governments of developing countries towards the enforcement of existing laws and national regulations, particularly the Basel Convention and the Bamako Convention, especially among the countries in the West African region, which is the major hub of trade in used electrical equipment and e-waste importation [68]. Stricter measures regulating the movement of e-waste substances from the UK, the USA and the EU member states should be put in place with developing states being able to prohibit or object to shipments of waste in order to implement the principles of proximity and self-sufficiency [69].

## Conclusion

The desire for technological advancement and alternative ICT-based options created to address the effect of the COVID-19 lockdown is changing settings in the developing world. The situation in developing countries is ‘not pretty’, according to Sullivan [68]. Indeed, it is a ticking time bomb that may explode soon, if not defused in time. The resort to ICT measures to minimise the effect of the lockdown and as an alternative to face to face activities is increasing and is likely to remain so even post-COVID-19, as some of these innovations are expected to remain complementary to traditional physical interactions. This will invariably lead to an increase in the importation of electrical and electronic devices and a consequential increase in the generation of e-waste.

Based on the above, there is a need for Nigeria and other developing countries to be cautious when embracing the increased reliance on technological innovations as an alternative to physical interactions as necessitated by the COVID-19 pandemic. Whilst the use of ICT options as a viable alternative to face-to-face interactions may not be a negative development during and post the COVID-19 pandemic, the existing frameworks are inadequate to manage the resultant increase in e-waste. There is a need for developing countries to not only start the conversation and plan for managing the looming increased e-waste situation but to start putting measures in place to minimise the generation of such waste and to ensure its proper disposal in an environmentally sound manner during and post COVID-19. The problems associated with informal recycling of e-waste will continue to spread in developing countries in Africa, Asia, and the Indian subcontinent with these countries increasingly becoming major receptacles for the developed world’s unwanted electronics and electrical equipment.

## Data Availability

Data sharing is not applicable to this article as no new data were created or analysed in this study.
